# Flexural behaviour of strengthened damaged steel beams using carbon fibre-reinforced polymer sheets

**DOI:** 10.1038/s41598-022-14471-9

**Published:** 2022-06-16

**Authors:** Wenyu Hou, Lianguang Wang, Di Shi

**Affiliations:** 1 School of Transportation and Geomatics Engineering, Shenyang Jianzhu University, Shenyang, 110168 China; 2 Jilin University of Architecture and Technology, Changchun, China; 3 Changchun University of Architecture and Civil Engineering, Changchun, China

**Keywords:** Civil engineering, Mechanical engineering

## Abstract

This paper presents the test and finite element analysis results of a study on the flexural behaviour of damaged steel beams strengthened with carbon fibre-reinforced polymer (CFRP) sheets. The test results showed that the yield load, ultimate load and elastic stiffness of the steel beams with 100% loss of tension flange were 68.3%, 73.8% and 13.5% greater than the yield load, ultimate load and elastic stiffness of the steel beam with 28% loss of the web after static loading. The yield load and elastic stiffness of the steel beam after overloading were 8.7% and 24.5% greater than the yield load and elastic stiffness of the steel beam without overloading. The damage level had a significant effect on the yield load, ultimate bearing capacity and elastic stiffness of the steel beams regardless of whether the steel beams were under static loading or overloading. The damaged steel beam could be repaired by CFRP sheets, the increased layers of CFRP sheets could improve the yield load, ultimate bearing capacity and elastic stiffness of the steel beams, and the strains of CFRP sheets would decrease because of overloading. The numerical analysis results showed that compared with the steel beam without overloading, the deflection and strains of the steel beam after overloading were much smaller. The yield load and the elastic stiffness increased with the increment of the overloading amplitude, and the increment of overloading number could reduce the yield load and elastic stiffness. All the yield loads of the steel beams after overloading were greater than the yield loads of the steel beams without overloading, but the ultimate bearing capacities were smaller.

## Introduction

One of the major challenges confronting the civil engineering community today is extending the service life of degraded steel structures. Replacement of degraded structures is often not feasible, and repairing them using conventional materials is inefficient in terms of the cost, social and environmental impacts and durability. Some structures are always used under overloading, in which the service load is greater than 70% of the ultimate load of the structure. In recent years, a new method to repair damaged steel structures has been to use carbon fibre-reinforced polymer (CFRP) sheets. CFRP sheets have unique material and mechanical properties, such as low self-weight, high strength and stiffness, and good durability. The CFRP sheets can be epoxy-bonded to the tension face of the damaged members to restore or enhance the ultimate bearing capacity of steel members. During the past years, there have been many studies on the repair and retrofitting of steel members with epoxy-bonded fibre-reinforced polymer (FRP) materials. Colombi and Poggi^1^ investigated an experimental and numerical program to characterize the static behaviour of steel beam reinforcement by pultruded CFRP strips. The main objective of the experimental programme was the evaluation of the force transfer mechanism, the increment of the load-carrying capacity and the bending stiffness. Use of the pultruded CFRP strips also allowed us to validate different analytical and numerical models for the static analysis of reinforced beams. Bocciarelli^2^ presented a simple approach to evaluate the response of statically determined steel beams reinforced by carbon fibre-reinforced polymer plates in the elastic–plastic regime. The solution proposed was valid only at a certain distance from the reinforcement ends where the response of the structure was not influenced by the local effects due to the abrupt termination of the reinforcement. Sugiura et al.^3^ presented the applicability of CFRP adhesion for the repair of corroded steel members. The peeling behaviour of CFRP was investigated experimentally in tensile and flexural tests of steel members with adhered CFRP. Based on the experimental results, the design method for determining the required volume and bonding length of CFRP and checking the peeling of CFRP from steel were given. Wu et al.^4^ investigated the fatigue behaviour of artificially notched steel beams strengthened with four different types of materials tested under equivalent tensile stiffness. The test results showed that the application of a fibre-reinforced composite plate could not only delay crack initiation, decrease the crack growth rate, and prolong the fatigue life but also reduce the stiffness decay and residual deflection. Yu et al.^5^ investigated the effectiveness of CFRP plates in extending the fatigue life of steel structures. The experimental results showed that the CFRP patches could effectively slow crack growth, prolonging the fatigue life and late strengthening at a larger damage level tended to result in a more significant extension in the remaining fatigue life. Bocciarelli and Colombi^6^ presented a simple approach to compute the elastoplastic response of a steel beam reinforced with a CFRP lamina. The main conclusion was that a reinforced section had to reach a large curvature to develop its ultimate bending moment resistance, and for this reason, it was necessary to use stiffeners to avoid local instability problems both in the web and in the flanges. Hmidan et al.^7^ reported the crack tip behaviour of wide-flange W4 × 13 steel beams strengthened with CFRP sheets. The results showed that the CFRP properties, such as the number of layers and modulus, influenced the crack tip plasticity of the strengthened beams. Colombi et al.^8^ executed fatigue tests on cracked steel plates (single edge specimens) reinforced by strips bonded to a single side. The results showed that CFRP materials bonded around the tip area extended the fatigue life of the damaged steel elements by a factor of approximately 3. Ghafoori and Motavalli^9^ experimentally and numerically studied the lateral-torsional buckling (LTB) of steel beams strengthened by normal modulus (NM) CFRP laminates. Increasing the prestress in the CFRP laminate was shown not to always increase the buckling strength of retrofitted slender steel beams. Wang et al.^10^ used CFRP sheets and prestressed CFRP sheets to repair steel–concrete composite girders. The results showed that CFRP sheets had no significant effect on the yield loads of strengthened composite girders but had a significant effect on the ultimate loads. Colombi and Fava^11^ investigated nine CFRP-strengthened cracked steel beams under fatigue loading. Experimental results revealed the presence of a debonded area between the reinforcement and the steel substrate at the crack location. Debonding clearly had a detrimental effect on the reinforcement effectiveness. Gholami et al.^12^ evaluated the performance of I-section steel beams strengthened with pultruded CFRP plates on the bottom flange after exposure to diverse conditions, including natural tropical climate, wet/dry cycles, plain water, salt water and acidic solution. The study found that the adhesive layer was the critical part, and the performance of the system related directly to the behaviour and the ductility of all strengthened beams increasing after exposure. Aljabar et al.^13^ extended the current knowledge of CFRP strengthening of steel elements in tensile fatigue loading to the case of mixed tension and shear loading. A shifting phenomenon was identified to describe the influence of the mixed mode in terms of crack propagation. A mixed mode modification factor was developed to estimate the fatigue life of CFRP-strengthened steel plates with inclined initial cracks. Hu et al.^14^ proposed fatigue design guides and programs for CFRP-strengthened steel structures. CFRP was shown to be effective for strengthening steel structures under fatigue. CFRP could extend the fatigue life under a certain loading condition or increase the allowable stress range when a certain fatigue life was desired. Yousefi et al.^15^ presented the findings of experimental and numerical investigations on failure analysis and structural behaviour of notched steel I-beams reinforced by bonded CFRP plates under static load. The results showed that the CFRP failure modes in strengthening of deficient steel I-beams included end-debonding, below-point load debonding, splitting and delamination. Bocciarelli et al.^16^ proposed analytical and numerical models of elastobrittle adhesives to evaluate the stress and strain distribution in the reinforcement at a given crack length. The experimental results were considered to validate the proposed numerical and analytical techniques. The calculated results were in good agreement with the experimental results. Martinelli et al.^17^ studied the bonding behaviour of fibre-reinforced polymer (FRP) composites glued to steel substrates by experimental and numerical simulations. The results showed that the incorporated bond-slip relationship in the proposed numerical model had a significant effect on the numerical results. Therefore, it was important to identify the realistic bond-slip relationships with different adhesive types and curing conditions (by conducting experimental tests). Zhang et al.^18^ investigated the flexural behaviour of corroded steel beams strengthened by CFRP plates. The effects of corrosion and prestressing force levels on the flexural capacity, failure modes and interfacial stress were investigated. The results showed that the failure mode of the corroded beams was the fracture of the CFRP plate after the shear failure of the interface on the midspan, and the fracture location of the CFRP plate was mostly at the loading point. The rough surface of corroded steel can enhance the efficiency of stress transfer at the interface, thereby improving the effective bond length of the interface. The shear stress was concentrated mainly on the CFRP plate end, and the peak value appeared at the loading point. Compared with the reference beam, the ultimate flexural capacity of the corroded beam strengthened by the CFRP plate with a 15% prestress level increased at a ratio of 21%, and the utilization ratio of the CFRP plates was up to 71.59%. Hu and Feng^19^ presented a design method for CFRP-reinforced damaged steel structures and developed a design program. The results showed that CFRP reinforcement can improve the usable life under a certain stress range and the allowable stress range under the premise of achieving the target usable life. Deng et al.^20^ studied the flexural fatigue performance of damaged steel beams strengthened by carbon fibre-reinforced plastic-optical fibre Bragg grating (CFRP-OFBG) plates. The test results showed that CFRP-OFBG plate reinforcement effectively reduced the fatigue crack growth rate of damaged steel beams and increased the fatigue life of damaged steel beams by 22.46%. The analysis and test results showed that the minimum error between the calculated value of the life prediction model and the test value was − 24.13%, and the maximum error was − 5.61%.

However, a few studies have discussed the use of epoxy-bonded plates or sheets to strengthen steel beams that have a tension flange or web defect such as a notch, especially applied overloading to these steel beams. In this paper, the tension flange or web was partly observed at the midspan of the steel beams. CFRP sheets were adhesively bonded to the lower side of the flange of steel beams to restore the bearing capacity and the elastic stiffness, and then the static loading or overloading was applied to these steel beams. The effects of the CFRP sheet layers, damage level of the steel beam and overloading number were investigated.

## Experimental program

**Figure 1:**
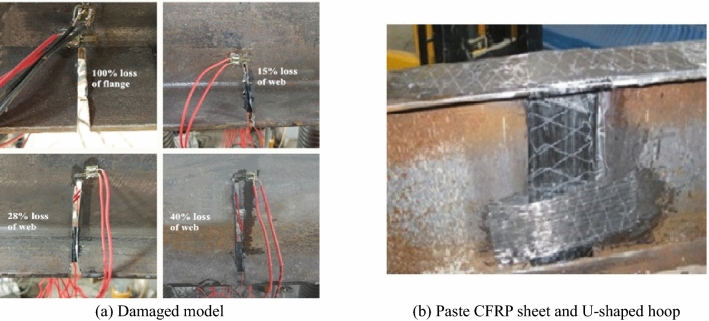
The preparation of specimens.

A total of seven artificially damaged steel beams were fabricated. The steel beams were made of typical China Standard steel I20A, of which the depth was 200 mm, the width of the flange was 100 mm, the thicknesses of the flange and web were 11.4 mm and 7 mm, respectively, and the area of the section was 3550 mm^2^. The steel sections were cut into 1.9-m-long beams, and four different damage levels of 100% loss of tension flange and 15%, 28%, 40% loss of tension web were cut at midspan of the steel beams, as shown in Fig. 1a. A tensile test of I-shaped steel was conducted. The yield strength and tensile strength of the I-shaped steel were 265 MPa and 442 MPa, respectively. The externally bonded strengthening systems selected for this test were high-strength CFRP sheets. The thickness of the CFRP sheets was 0.167 mm, the width was 60 mm, and the length was 1,500 mm. A tensile test of CFRP sheets was conducted as documented in reference^21^, and the average tensile strength was 3456 MPa; the elastic modulus was 258 GPa. The resin adhesive used to paste the CFRP sheet was matched with the CFRP sheet, and its shear strength was 19.4 The CFRP sheet was pasted in the same way as documented in reference^21^. CFRP sheets were pasted on the bottom of the tension flange, and then U-shaped hoops were pasted at the end of the CFRP sheets to ensure that the CFRP sheets could be anchored to the steel beams, as shown in Fig. 1b. The detailed parameters of the steel beams are given in Table 1.

**Table 1:** Test matrix and results.

Specimen number	Damage level	Number of CFRP layers	Overloading amplitude	Overloading number	Yield load of bottom flange P_t_(kN)/(%P_u_)	Yield load of top flange P_y_ (kN)/(%P_u_)	Ultimate load P_u_ (kN)	Failure modes (CFRP sheet)
SB0	100% (flange)	0	–	–	57.7/(32.5)	124.6/(70.3)	177.3	–
FSB1	28% (web)	One	–	–	34.1/(30.3)	81.4/(72.4)	112.5	Rupture
FSB2	100% (flange)	One	0.7 P_u_	100	108.1/(58.4)	148.9/(80.4)	185.1	Debonding
FSB3	15% (web)	One	0.7 P_u_	100	47.9/(34.2)	120/(85.6)	140.2	Debonding
FSB4	100% (flange)	One	–	–	66.8/(34.2)	137/(70.1)	195.5	Rupture
FSB5	40% (web)	One	0.7 P_u_	100	28/(37.1)	56.8/(75.2)	75.5	Rupture
FSB6	40% (web)	Two	0.7 P_u_	100	27.5/(29.2)	78.6/(83.3)	94.3	Rupture

## Experimental setup

**Figure 2:**
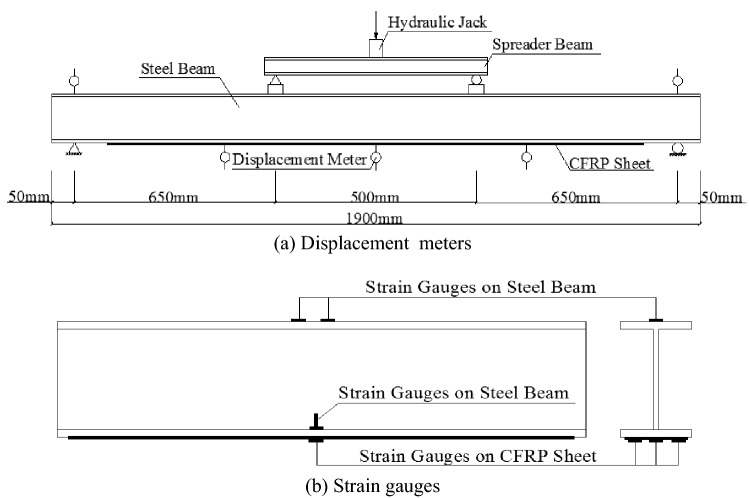
Schematic of locations of the displacement meters and strain gauges at beam.

**Figure 3:**
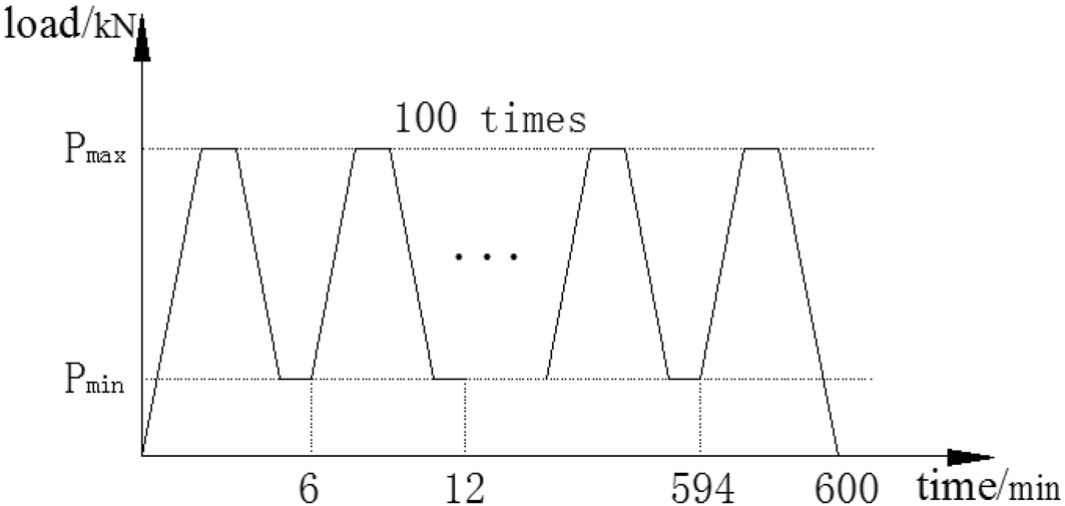
Overloading process.

The steel beams were loaded in four-point bending with 500-mm spacing between two concentrated point loads and with two equal shear spans of 650 mm, as shown in Fig. 2. Rubber bearings were used at the supports. Loading was applied across the full width of the top flange of the steel beam by a spreader beam that was placed between the rubber bearings on the top of the steel beam. Four-point bending tests were performed using hydraulic jacks, as shown in Fig. 2a. The overloading process needed to set the minimum load (P_min_), maximum load (P_max_) and the number of the overloads. Then, the steel beams would be cycled in this range. A time period of one cycle was approximately 6 min, as shown in Fig. 3. After overloading, the steel beams were subjected to loading until they were disrupted. To observe the behaviour of the steel beams under investigation, the strains, loads and deflections were measured at the desired locations. The strains were measured by electrical resistance strain gauges that were placed on the top of the top flange, the bottom flange or the web near the notch and the midspan of the CFRP sheets, as shown in Fig. 2b. Five displacement meters were mounted at the end and midspan of the beams, and the meters were used to measure the vertical deflection, as shown in Fig. 2a.

## Test results and discussion

### Failure modes

As indicated previously, the steel beams strengthened with adhesively bonded CFRP sheets could display four distinct failure modes: the bottom flange being crippled; CFRP sheet rupture with the web being crippled; CFRP sheet debonding with the web being crippled; and CFRP sheet rupture with the bottom flange being crippled.

CFRP rupture with the web crippling was the dominant failure mode in the steel beams without overloading. The rupture of the CFRP sheets was sudden, and there was no sign of bonding failure between the CFRP sheet and the steel flange in specimens FSB1 and FSB4. Because the damage level was small in specimen FSB4, only the bottom flange was crippled. The failure mode of SB0 was that the bottom flange was crippled because there was no CFRP sheet bonded.

The failure modes of the steel beams under overloading were CFRP sheet rupture with the web being crippled and CFRP sheet debonding with the web being crippled. CFRP debonding appeared in specimens FSB2 and FSB3. CFRP rupture appeared in specimens FSB5 and FSB6. All webs of steel beams were crippled when the CFRP sheets debonded or ruptured.

**Figure 4:**
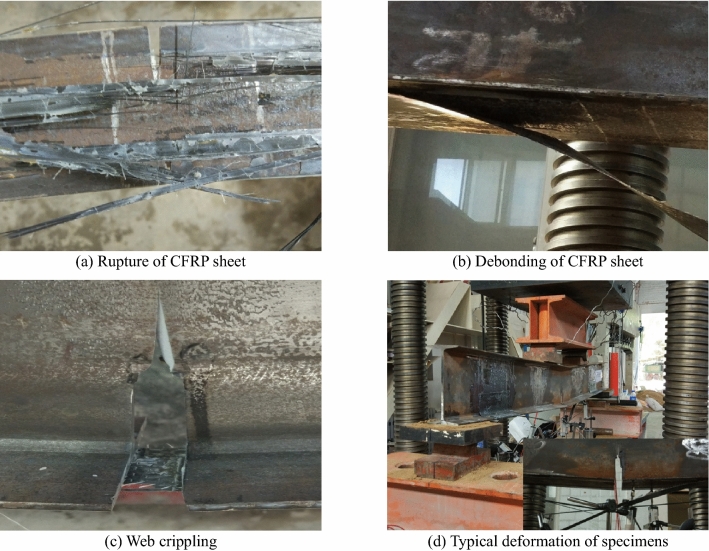
Failure modes of test specimens.

The experimental results revealed that ultimate failure was usually accompanied by large deflection and that the web or bottom flange was crippled. Both the web and flanges of the steel beams yielded, as shown in Fig. 4.

### Load–deflection curves of the cyclic stage

**Figure 5:**
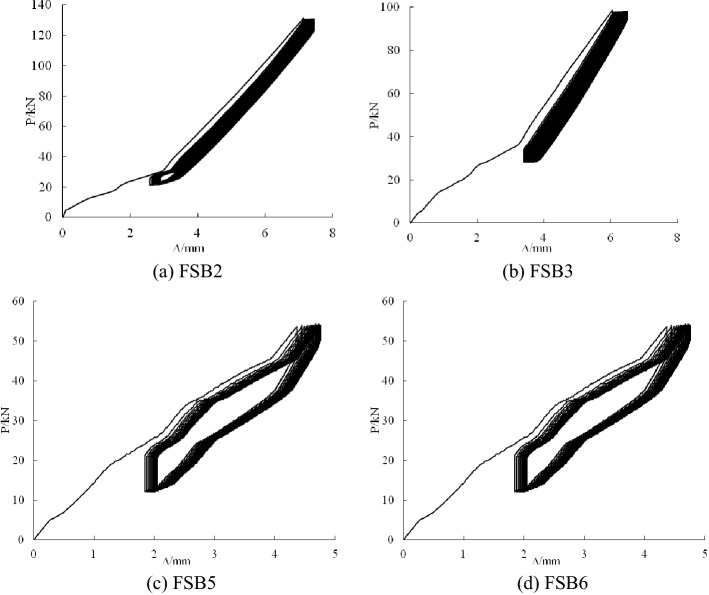
Load–deflection relationship of specimens with 100 cycles.

Figure 5 shows the load–deflection curves at the cyclic stage. In the unloading process of FSB2, FSB3, FSB5 and FSB6 after 100 cycles, the curves did not directly decline according to the original curves. This condition showed that the overloading amplitude exceeded the critical value of the elastic stage of the steel beam, and all of the steel beams were in the elastic–plastic stage. The load–deflection curves of FSB2 and FSB3 were smoother than the load–deflection curves of FSB5 and FSB6 due to the large notches of FSB5 and FSB6. The recovery capacity of FSB5 and FSB6 was reduced, resulting in an uneven curve.

### Effect of CFRP sheet

**Figure 6:**
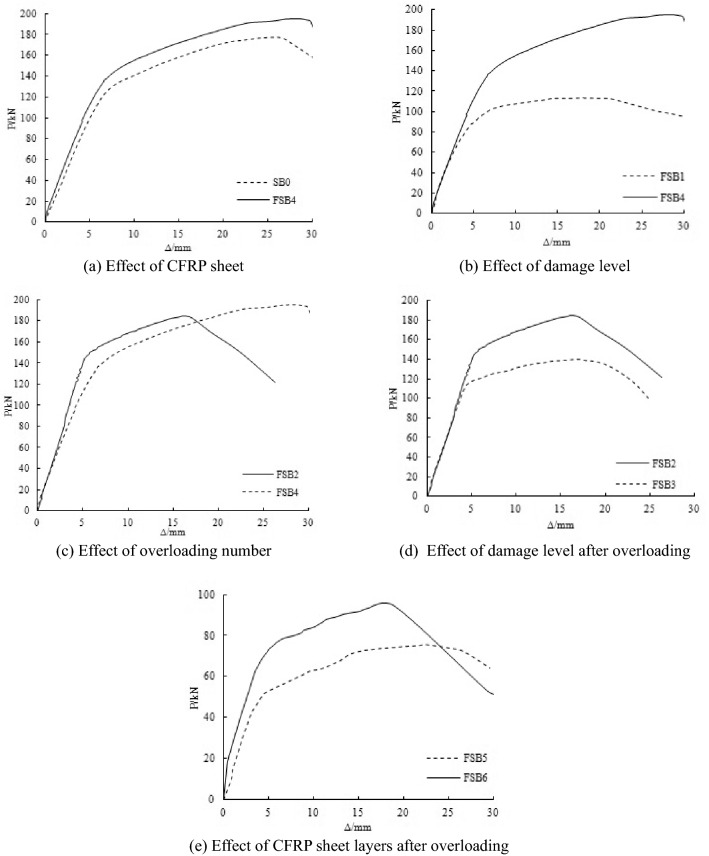
Load–deflection curves of strengthened steel beams.

Figure 6a shows the load–deflection curve of the steel beams with 100% flange damage. SB0 was the steel beam that was not strengthened, and FSB4 was the steel beam strengthened with one layer of CFRP sheet. Both of the specimens underwent only static loading. The yield load of FSB4 was 137 kN, which was 10% greater than the yield load of SB0, and the elastic stiffness was 14.5% greater than the elastic stiffness of SB0. The ultimate load of FSB4 was 195.46 kN, which was 10.3% greater than the ultimate load of SB0. The results show that the epoxy-bonded CFRP sheet significantly increased the yield load, ultimate bearing capacity and elastic stiffness of the steel beam.

### Effect of damage level

Figure 6b shows the load–deflection curve of the steel beams with different damage levels, which were both strengthened with one layer of CFRP sheet. The damage levels of 100% loss of tension flange and 28% loss of web were cut at midspan of steel beams in FSB4 and FSB1, respectively. Both the specimens underwent only static loading. When the load did not reach 65% Pu, the elastic stiffness of the two steel beams was the same. The yield load and ultimate load of FSB4 were 68.3% and 73.8% greater than the yield load and ultimate load of FSB1, and the elastic stiffness was 13.5% greater than the elastic stiffness of FSB1. The yield load and ultimate load obviously changed as a result of the damage level. Compared with the yield load and ultimate load, the damage level had no significant effect on the elastic stiffness.

### Effect of overloading number

Figure 6c shows the load–deflection curve of the steel beams with 100% flange damage after overloading. FSB4 was the steel beam with only static loading. FSB2 was the steel beam with 100 times of 0.7 Pu overloading and an entirely static loading. When the load did not reach 60 kN, the elastic stiffness of the two steel beams was the same. The yield load of FSB2 was 148.9 kN, which was 8.7% greater than the yield load of FSB4, and the elastic stiffness was 24.5% greater than the elastic stiffness of FSB4. The ultimate load of FSB2 was 185.08 kN, which was 5.3% smaller than the ultimate load of FSB4. The results show that overloading could influence the yield load and the elastic stiffness. The yield load and the elastic stiffness increased with the increment of the number of overloading because of the cold hardening of steel, but the ultimate load would be reduced.

### Effect of damage level after overloading

Figure 6d shows the load–deflection curve of the steel beams with different damage levels after overloading. The damage levels of 100% loss of tension flange and 15% loss of web were cut at midspan of steel beams in FSB2 and FSB3, respectively. The steel beams were subjected to 100 times of 0.7 Pu overloading and an entirely static loading. When the load did not reach 75% Pu, the elastic stiffness of the two steel beams was the same. The yield load of FSB2 was 24.1% greater than the yield load of FSB3. The ultimate load of FSB2 was 32% greater than the ultimate load of FSB3.The yield load and ultimate bearing capacity of strengthened steel beams has been shown to change as a result of damage levels after overloading.

### Effect of the number of CFRP sheet layers after overloading

Figure 6e shows the load–deflection curve of the steel beams with 40% web damage strengthened by different numbers of layers of CFRP sheets after overloading. FSB5 was repaired with one layer of CFRP sheet, and FSB6 was repaired with two layers of CFRP sheets. The steel beams were subjected to 100 times of 0.7 Pu overloading and an entirely static loading. The yield load of FSB6 was 78.6 kN, which was 38.4% greater than the yield load of FSB5, and the elastic stiffness was 36.9% greater than the elastic stiffness of FSB5. The ultimate load of FSB6 was 94.32 kN, which was 24.9% greater than the ultimate load of FSB5. The results show that the increment of CFRP sheet layers could improve the yield load, elastic stiffness and ultimate bearing capacity of the steel beams after overloading.

### Load–deflection curves before and after overloading

**Figure 7:**
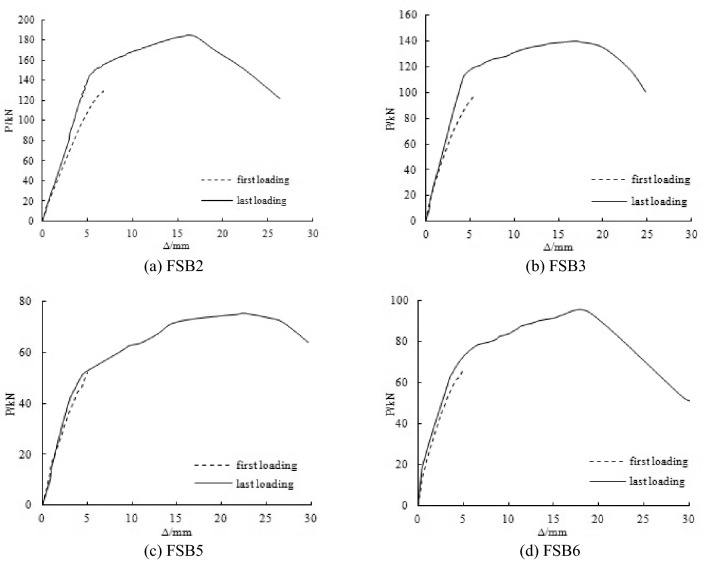
Load–deflection curve of strengthened steel beams under loading.

Figure 7 shows the load–deflection curves of the first and last loadings of FSB2, FSB3, FSB5 and FSB6. After overloading, the elastic stiffness of all the steel beams increased. The elastic stiffness of FSB2, FSB3, FSB5 and FSB6 of the last loading was 31.1%, 23.2%, 14% and 15% greater than the elastic stiffness of the first loading, respectively. The elastic stiffness of the steel beams with large damage levels increased less than the elastic stiffness of the steel beams with small damage levels. The elastic stiffness of the steel beams was shown to be able to be increased after overloading because of the cold hardening of the steel. However, the effect of cold hardening on the steel beam decreased with the incremented damage level of the steel beam.

### Load-strain curves

**Figure 8:**
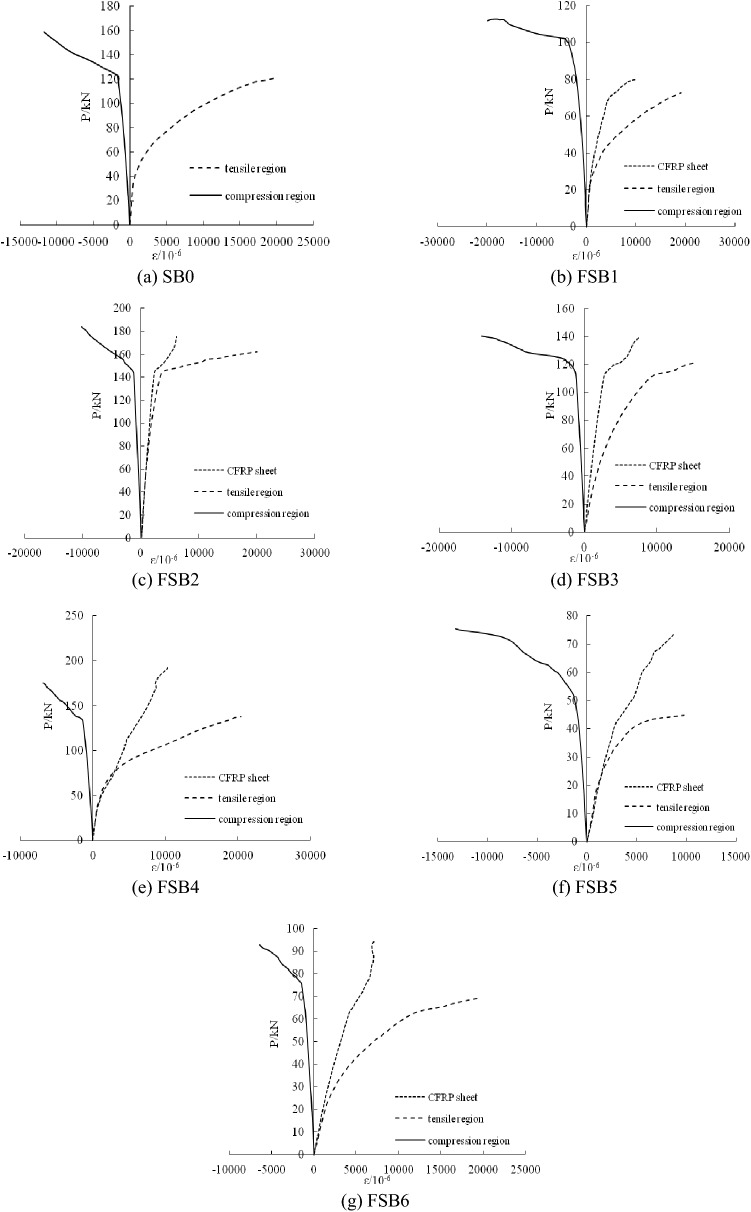
Load-strain curves of strengthened steel beams.

At the beginning of the test, the load-strain curves of the strengthened steel beams were linear, as shown in Fig. 8. The lines represent the strains in the tensile region of steel beams, the compression region of steel beams and the CFRP sheets. The tensile region of the steel beams started to yield at approximately 30 to 35% of the ultimate load. However, because of the effect of cold hardening, the FSB2 specimen yielded at 58% of the ultimate load. After yielding, the effectiveness of the CFRP sheets was much better. The strains in the tension region of the strengthened steel beams decreased significantly. At the same load level, the strains of the steel beam with a large damage level were greater than the strains of the other steel beams. When the load reached approximately 70 to 85% of the ultimate load, the compression region of the steel beams started to yield. Then, the compressive strains on the compression region of the steel beams became nonlinear. The strains of the CFRP sheets were approximately 10,000 με after static loading and were approximately 7500 με after overloading. The results show that the damage levels of steel beams could affect the strains of the strengthened steel beams. After overloading, the yield load of the steel beam increased, but overloading had no significant effect on the steel beams with large damage levels. The number of CFRP sheet layers could have an effect on the yield load and ultimate bearing capacity. The strains of the CFRP sheets would decrease because of the overloading.

## Numerical analysis

### Model development

The FE analysis was performed using ABAQUS. All the steel beams were modelled. The geometry and loading arrangements of the model were adopted in accordance with the tested beams. The end support was modelled using a roller support that restrained the vertical movement of the beam. The longitudinal translation of the beam was allowed. A tie constraint was employed for the bonds between the steel beam and CFRP sheet because the interface slip was not considered in this model.

**Figure 9:**
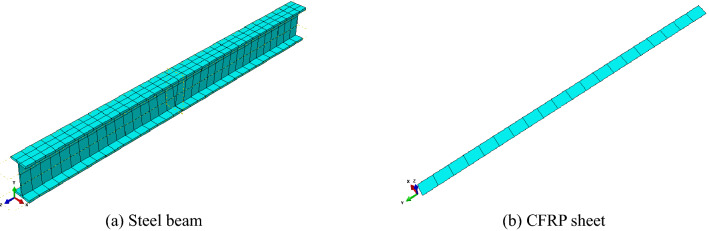
Mesh generation.

**Figure 10:**
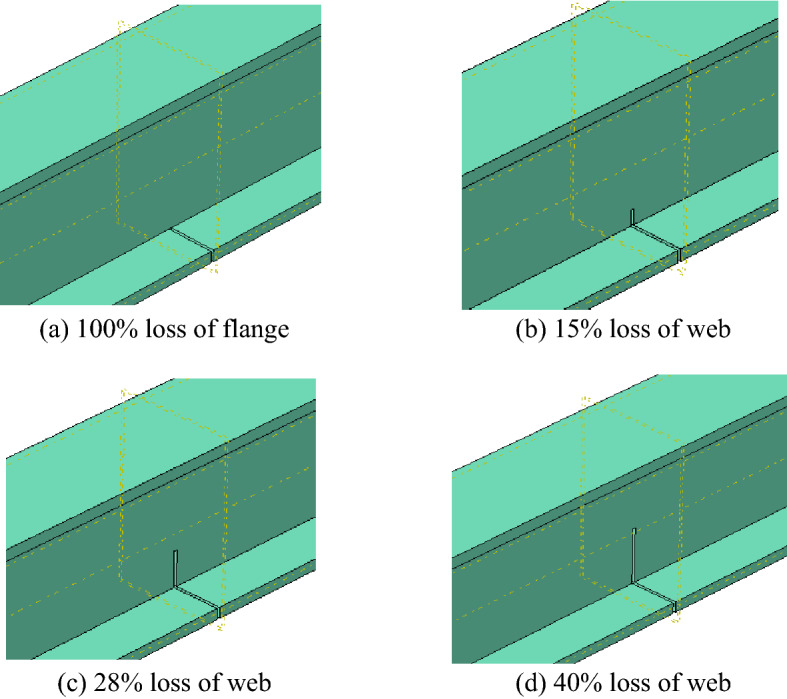
Damage model.

The steel beam was modelled as a C3D8R finite element (eight-node solid finite elements with reduced integration). The CFRP sheet was modelled as an SR4 finite element (four-node shell finite elements with reduced integration). Element sizes were adopted based on the mesh discretisation study. The finite element mesh used for the analysis is shown in Fig. 9. There were 4 damage models in the finite element analysis, as shown in Fig. 10.

### Material properties and constitutive models

#### Steel beam

**Figure 11:**
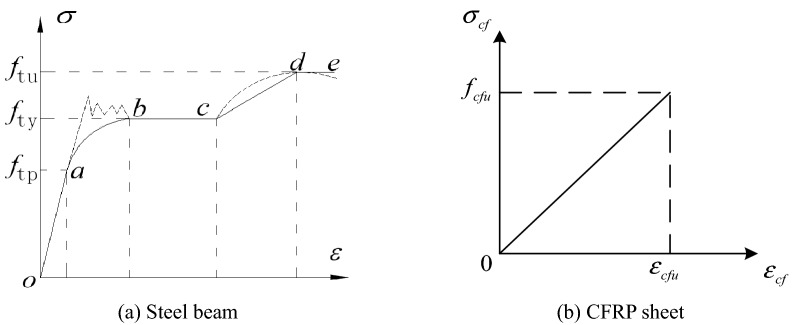
Stress–strain relationship.

The steel was assumed to be an elastoplastic with hardening and saturation material and identical in tension and compression, as shown in Fig. 11a. The stress–strain relationship for steel is shown in Eq. (1). A Poisson’s ratio of 0.3 was used for the steel beam, as shown in Table 2.

1 $$ \sigma  = \left\{ {\begin{array}{*{20}l}    {E_{{\text{t}}} \varepsilon _{{\text{t}}} } \hfill & {(\varepsilon _{{\text{t}}}  \le \varepsilon _{{{\text{te}}}} )} \hfill  \\    { - A\varepsilon _{{\text{t}}}^{{\text{2}}}  + B\varepsilon _{{\text{t}}}  + C} \hfill & {(\varepsilon _{{{\text{te}}}}  < \varepsilon _{t}  \le \varepsilon _{{{\text{te1}}}} )} \hfill  \\    {f_{{{\text{ty}}}} } \hfill & {(\varepsilon _{{{\text{te1}}}}  < \varepsilon _{t}  \le \varepsilon _{{{\text{te2}}}} )} \hfill  \\    {f_{{{\text{ty}}}} \left[ {{\text{1}} + {\text{0}}{\text{.6}}\frac{{\varepsilon _{{\text{t}}}  - \varepsilon _{{{\text{e2}}}} }}{{\varepsilon _{{{\text{e3}}}}  - \varepsilon _{{{\text{e2}}}} }}} \right]} \hfill & {(\varepsilon _{{{\text{te2}}}}  < \varepsilon _{{\text{t}}}  \le \varepsilon _{{{\text{te3}}}} )} \hfill  \\    {{\text{1}}{\text{.6}}f_{{{\text{ty}}}} } \hfill & {(\varepsilon _{{\text{t}}}  > \varepsilon _{{{\text{te3}}}} )} \hfill  \\   \end{array} } \right. $$

where $A = \frac{{{0}{\text{.2}}f_{{{\text{ty}}}} }}{{(\varepsilon_{{{\text{te1}}}} - \varepsilon )^{{2}} }}$, $B = 2A\varepsilon_{{{\text{te1}}}}$, $C = 0.8f_{{{\text{ty}}}} + A\left( {\varepsilon_{{{\text{te}}}} } \right)^{{2}} - B\varepsilon_{{{\text{te}}}}$, $\varepsilon_{{{\text{te}}}} = 0.8f_{{{\text{ty}}}} /E_{{\text{t}}}$,$\varepsilon_{{{\text{te1}}}} = {1}.{5}\varepsilon_{{{\text{te}}}}$, $\varepsilon_{{{\text{te2}}}} = {10}\varepsilon_{{{\text{te}}}}$, $\varepsilon_{{{\text{te3}}}} = {100}\varepsilon_{{{\text{te}}}}$, $E_{{\text{t}}}$ is the elastic modulus of steel, $f_{{{\text{ty}}}}$ is the yield strength of steel, and $f_{{{\text{tu}}}}$ is the ultimate strength of steel.

**Table 2:** Material properties.

	Element type	Material	Poisson’s ratio
Steel beam	C3D8R	Elasto-plastic with hardening and saturation	0.3
CFRP sheet	SR4	Linear elastic orthotropic	–

#### CFRP sheet

The CFRP was modelled as a linear elastic orthotropic material, as shown in Fig. 11b, and the relationship of stress and strain was:

2 $$ \left\{ {\begin{array}{*{20}l} {\sigma_{cf} = E_{cf} \varepsilon_{cf} \begin{array}{*{20}c} {} & {0 \le } \\ \end{array} \varepsilon_{cf} \le \varepsilon_{cfu} } \\ {\sigma_{cf} = 0\begin{array}{*{20}c} {} & {} & {\varepsilon_{cfu} \le } \\ \end{array} \varepsilon_{cf} } \\ \end{array} } \right. $$

where $\varepsilon_{cf}$ is the strain of the CFRP sheet, $\sigma_{cf}$ is the stress of the CFRP sheet, $\varepsilon_{cfu}$ is the allowable ultimate strain of the CFRP sheet, and $E_{cf}$ is the elastic modulus of the CFRP sheet.

### Results and discussions

#### Stress analysis

**Figure 12:**
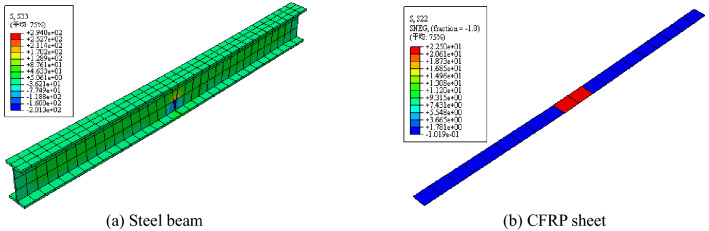
Stress images after overloading.

**Figure 13:**
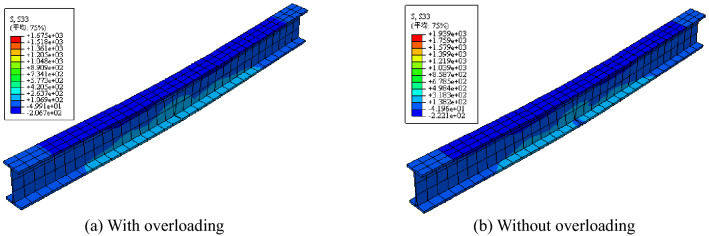
Stress comparison of steel beam with or without overloading.

**Figure 14:**
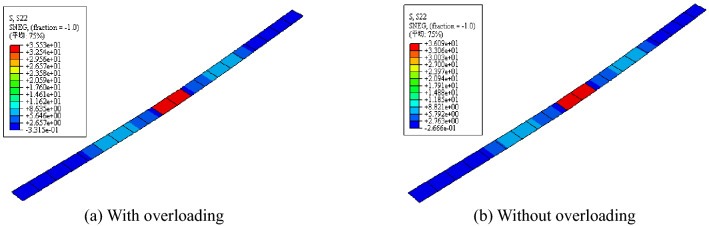
Stress comparison of CFRP sheet with or without overloading.

The stress images of the steel beam and CFRP sheet are shown in Figs. 12, 13, and 14. After overloading, the stress concentration appeared on the notch of the steel beam and midspan of the CFRP sheet. Compared with the steel beam without overloading, the strains of the steel beam and CFRP sheet were both smaller. Overloading is shown to be able to affect the final strain of the steel beam, and overloading would decrease the utilization of the CFRP sheet.

#### Deflection

**Figure 15:**
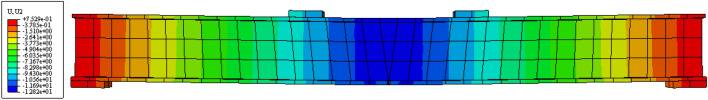
Residual deflection after overloading.

**Figure 16:**
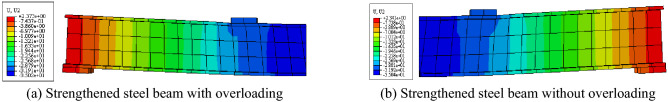
Deflection comparison of steel beam with or without overloading.

Figures 15 and 16 show the deflection images of the steel beam with and without overloading. After overloading, residual deflection occurred in the steel beam. The value of residual deflection was related to the overloading number and overloading amplitude. The deflection of the steel beam after overloading was smaller than the deflection of the steel beam after static loading.

#### Comparison between test results and finite element calculation results

**Figure 17:**
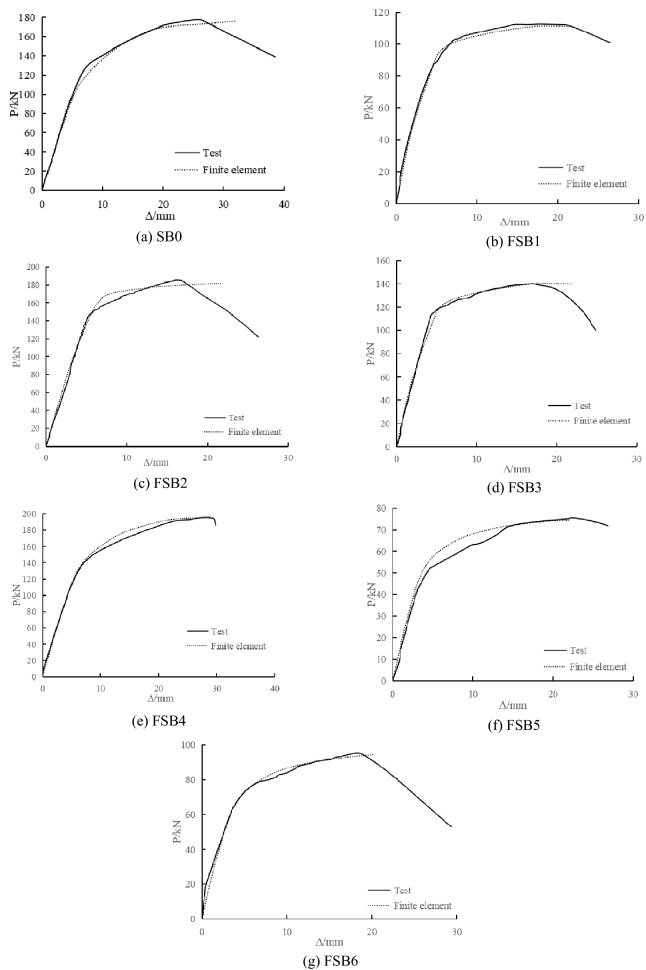
Comparison between test and finite element calculation of load–deflection curves.

**Figure 18:**
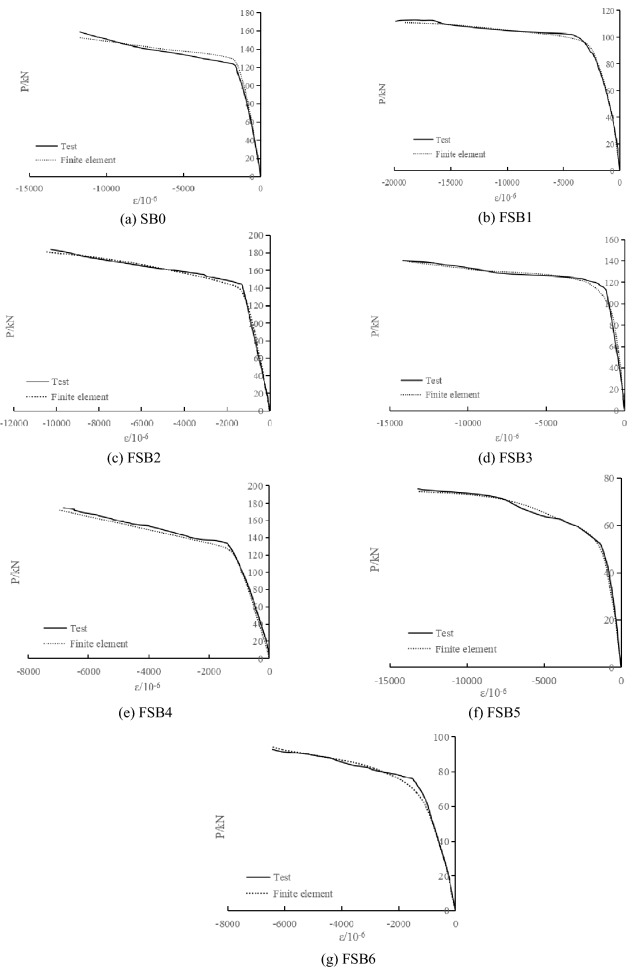
Comparison between test and finite element calculation of load-strain curves (compression strain).

Figures 17 and 18 show a comparison between the test results and finite element calculation results. The finite element calculation results were in good agreement with the test results, indicating that the calculation model of the damaged steel beams strengthened with CFRP sheets was correct.

### Parameter analysis

#### Effect of overloading number

**Figure 19:**
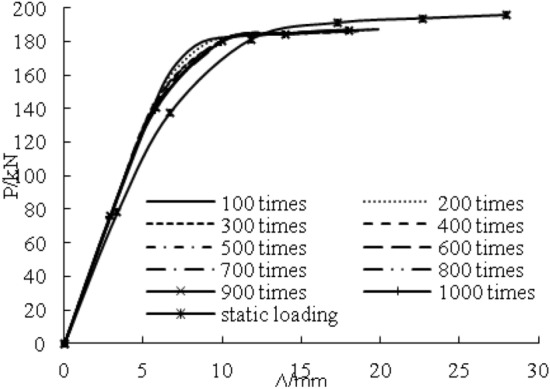
Effect of overloading number.

Figure 19 shows the load–deflection curves of the steel beams with 100% flange damage under different overloading numbers. The elastic stiffness of steel beams with 100–1000 overloading cycles was much larger than the elastic stiffness of steel beams with only static loading cycles. However, there was little change among all of them. The yield loads of steel beams with 100–1000 overloading cycles were 13.5%, 12.6%, 11.2%, 10.1%, 9%, 7.9%, 7%, and 6% greater than the yield loads of steel beams with only static loading. The ultimate loads of steel beams with 100–1000 overloading times were all smaller than the ultimate loads of steel beams with only static loading. The yield load and the elastic stiffness increased with the increment of the number of overloadings because of the cold hardening of steel. However, the improvement decreases as the overloading number increases. The ultimate load would be reduced because of the cold hardening of steel. The overloading number affected the ultimate load of the overloading beams.

#### Effect of overloading amplitude

**Figure 20:**
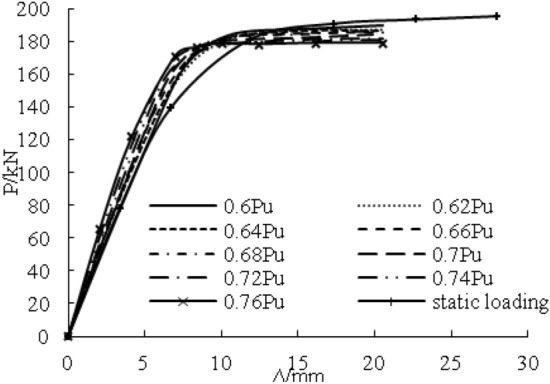
Effect of overloading amplitude.

Figure 20 shows the load–deflection curve of the steel beams with 100% flange damage under different overloading amplitudes. The elastic stiffness of steel beams with 0.6 Pu-0.76 Pu overloading amplitude were, respectively 6.2%, 6.5%, 8.6%, 11.2%, 14.1%, 15.8%, 16.5%, 18.4%, 19.8% greater than the elastic stiffness of steel beam with only static loading, and the yield loads were, respectively 15.8%, 17.9%, 21.1%, 22.8%, 25%, 26.8%, 27%, 28.9%, 30.5% greater than the yield loads of steel beam with only static loading. The ultimate loads were 1.9%, 2.3%, 2.9%, 3.2%, 4%, 4.7%, 5.7%, 6.8%, and 7.7% smaller than the ultimate loads of the steel beam with only static loading. The overloading amplitude increased per 0.02 Pu, and the yield load increased by approximately 2 kN, but the ultimate load decreased by approximately 1 kN. When the overloading amplitude reached 0.77 Pu, the strengthened steel beam was broken in the overloading stage.

## Conclusions

The findings of this study showed that adhesively bonded CFRP sheets can be effectively used to strengthen damaged steel beams and that overloading could affect the yield load and elastic stiffness of the steel beams. The following conclusions were drawn.

- All the steel beams after overloading could increase the yield load and elastic stiffness because of the cold hardening of steel after overloading. However, the larger the damage level of the steel beam was, the less the increment of the yield load and elastic stiffness would be. The yield load and elastic stiffness of the steel beam after overloading were 8.7% and 24.5% greater than the yield load and elastic stiffness of the steel beam without overloading. The ultimate load of the steel beam after overloading was 5.3% smaller than the ultimate load of the steel beam without overloading.
- The yield load, ultimate load and elastic stiffness of the steel beams with 100% loss of tension flange were 68.3%, 73.8% and 13.5% greater than the steel beam with 28% loss of the web after static loading. The yield load and ultimate load of the steel beam with 100% loss of tension flange were 24.1% and 32% greater than the steel beam with 15% loss of the web after overloading. Regardless of static loading or overloading, the damage level had a significant effect on the yield load and ultimate bearing capacity of the steel beams.
- The steel beam strengthened with CFRP sheets could increase the yield load, ultimate load and elastic stiffness by 10%, 10.3% and 14.5%, respectively, compared to the steel beam without strengthening after static loading. The steel beam strengthened with two layers of CFRP sheets increased the yield load, ultimate load and elastic stiffness by 38.4%, 24.9% and 36.9%, respectively, compared with the steel beam strengthened with one layer of CFRP sheet after overloading. The damaged steel beam was shown to be able to be strengthened by CFRP sheets, and the increased layers of CFRP sheets were shown to be able to improve the yield load, ultimate bearing capacity and elastic stiffness of the steel beams.
- The CFRP strains of the steel beams strengthened by CFRP sheets were approximately 10,000 με after static loading and approximately 7500 με after overloading. The strains of the CFRP sheets were shown to decrease because of overloading.
- Compared with the strengthened steel beam without overloading, the deflection and strains of the strengthened steel beam with overloading were much smaller. Because of the cold hardening of steel, the yield load and the elastic stiffness increased with the increment of overloading amplitude, and the increment of overloading number could reduce the yield load and elastic stiffness, but the ultimate load would be reduced.
